# Highly Pathogenic Avian Influenza A(H5N1) Viruses from Multispecies Outbreak, Argentina, August 2023

**DOI:** 10.3201/eid3004.231725

**Published:** 2024-04

**Authors:** Agustina Rimondi, Ralph E.T. Vanstreels, Valeria Olivera, Agustina Donini, Martina Miqueo Lauriente, Marcela M. Uhart

**Affiliations:** Robert Koch Institute, Berlin, Germany (A. Rimondi);; Instituto Nacional de Tecnología Agropecuaria Instituto de Virología e Innovaciones Tecnológicas, Buenos Aires, Argentina (A. Rimondi, V. Olivera);; University of California School of Veterinary Medicine, Davis, California, USA (R.E.T. Vanstreels, M.M. Uhart);; Southern Right Whale Health Monitoring Program, Puerto Madryn, Argentina (A. Donini, M.M. Uhart);; Secretaria de Ambiente y Cambio Climático de Provincia de Río Negro, Viedma, Argentina (M. Miqueo Lauriente)

**Keywords:** influenza, highly pathogenic avian influenza virus H5N1, HPAI H5N1, mammals, South America, Argentina, mammalian adaptation, pinnipeds, seabirds, transmission, viruses

## Abstract

We report full-genome characterization of highly pathogenic avian influenza A(H5N1) clade 2.3.4.4b virus from an outbreak among sea lions (August 2023) in Argentina and possible spillover to fur seals and terns. Mammalian adaptation mutations in virus isolated from marine mammals and a human in Chile were detected in mammalian and avian hosts.

In February 2023, the first case of highly pathogenic avian influenza (HPAI) A(H5N1) in Argentina was detected in a wild goose near the border with Bolivia and Chile (Appendix Figure 1) (*1*). In contrast with Peru and Chile, where extensive mortality of seabirds and marine mammals had been attributed to the virus in the preceding months (*2*,*3*), the initial spread of HPAI H5N1 in Argentina was largely limited to backyard and industrial poultry (94 outbreaks), causing the death or disposal of 2.2 million birds. Argentina declared itself free from the disease in poultry on August 8, 2023; before then, HPAI H5N1 detections in wildlife in Argentina had been scarce (7 events during February–April) and limited to aquatic birds (Anatidae, Laridae, and Rallidae) (*1*,*4*). However, soon thereafter, the national animal health services confirmed HPAI H5N1 in South American sea lions (*Otaria byronia*) from Río Grande, southernmost Argentina. Over subsequent weeks, the virus was detected in sea lions northward along the Argentina coast, and sporadic cases also occurred in South American fur seals (*Arctocephalus australis*). The most affected site was Punta Bermeja (Appendix Figure 1), the largest sea lion colony in Argentina, where an estimated 811 sea lions died over 2 months; minimal numbers (<5) of fur seals and terns were also affected (*1*,*4*). 

In collaboration with provincial authorities and park rangers, we collected swab samples (oronasal, rectal, tracheal, lung, and brain) from 16 deceased sea lions, 1 fur seal, 1 great grebe (*Podiceps major*), and 1 South American tern (*Sterna hirundinacea*) discovered at Punta Bermeja on August 26, 2023. A sampled adult male sea lion was seen alive showing clinical signs consistent with HPAI infection (inability to stand or walk, muscular tremors and spasms, difficulty breathing, and abundant oral mucus). We tested the samples by real-time reverse transcription PCR targeting influenza A virus (*5*) and confirmed that all were positive. On the basis of viral RNA yields, we selected brain samples from 4 sea lions, 1 fur seal, and 1 tern for full-genome sequencing (Appendix Figure 2). We used maximum-likelihood tree phylogenetic analysis (*6*) and mutational analysis to compare the sequences (GenBank accession nos. OR987081–128) with representative HPAI H5N1 strains from South America.

Phylogenetic trees (Figure; Appendix Figure 2) showed that the viruses we identified belong to HPAI H5N1 clade 2.3.4.4b and are closely related to H5N1 viruses that circulated in South America during 2022–2023. Our finding supports the hypothesis that, after introduction from North America into Peru in November 2022, HPAI H5N1 viruses continued spreading across the continent and into Argentina. Of note, the viruses from Punta Bermeja did not cluster with the hemagglutinin and neuraminidase sequences available from HPAI H5N1 first detected in a wild goose in Argentina. Instead, all gene segments from the viruses were closely related to virus sequences from sea lions in Chile and Peru (*2*; C. Pardo Roa, unpub. data, https://www.biorxiv.org/content/10.1101/2023.06.30.547205v); 6 gene segments (all except polymerase basic protein 1 and nucleocapsid protein) also clustered with the virus isolated from a human in Chile (*7*). That finding suggests that viruses from Punta Bermeja may have been derived from a separate HPAI H5N1 introduction into Argentina. Because of the lack of genomic data for HPAI H5N1 viruses circulating in Argentina during February–July 2023, the finer scale pathways (local geographic routes and host species involved) of how these viruses arrived at Punta Bermeja remain unclear. Even so, the viruses that we report did not cluster with those from birds in Uruguay, Brazil, or Bird Island (Antarctica), possibly suggesting separate pathways of virus spread.

**Figure:**
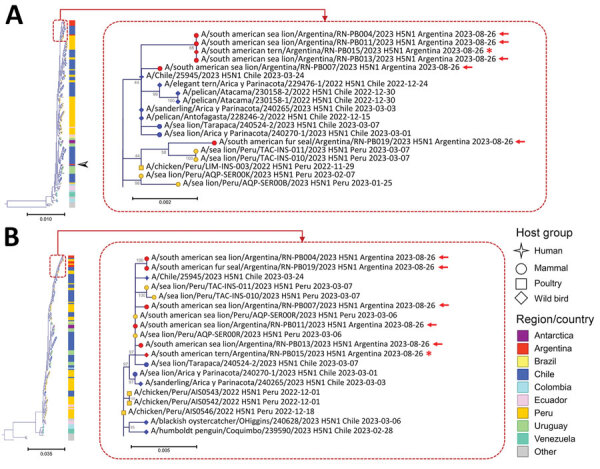
Maximum-likelihood trees for hemagglutinin (A) and polymerase basic 2 (B) gene segments evaluated in study of highly pathogenic avian influenza A(H5N1) in Argentina compared with reference strains from other countries in South America. Tree areas have been enlarged at right to show detail. Red arrows indicate virus from marine mammals in Argentina; red asterisk indicates virus from a tern in Argentina. Black arrowhead along full tree in panel A indicates the hemagglutinin sequence from the first detection of HPAI H5N1 in a wild goose in Argentina. Node shape represents host group, and node color (and bars adjacent to trees) represents the region/country. Branch lengths are drawn proportionally to the extent of changes. Values adjacent to nodes represent bootstrap support >40. Scale bars indicate nucleotide substitutions per site.

On the basis of previous comparisons with HPAI H5N1 isolates from other countries in South America, we identified 9 mutations already present in viruses infecting sea lions in Peru and Chile but not in the goose/Guangdong reference strain or in viruses from birds and mammals from North America in 2022 (Table). Specifically, we found Q591K and D701N mutations in polymerase basic 2 associated with increased pathogenicity to mammals (*8*). The virus we detected in the South American tern also has those mutations, but they were absent from previously reported HPAI H5N1 viruses from avian hosts in South America (except for A/sanderling/Arica y Parinacota/240265/2023, which has the D701N mutation). That finding further supports the hypothesis that HPAI H5N1 viruses from sea lions from Peru and Chile acquired mammalian adaptation mutations that improved their ability to infect pinnipeds while possibly retaining the ability to infect avian hosts. Given the rapid and widespread dissemination of the viruses among pinnipeds in South America and the substantial associated mortalities (*3*,*9*), it seems likely that pinniped-to-pinniped transmission played a role in the spread of the mammal-adapted HPAI H5N1 viruses in the region. It is alarming that the HPAI H5N1 viruses infecting pinnipeds and seabirds in Argentina share the same mammalian adaptation mutations as the virus from the affected human in Chile, which highlights the potential threat posed by these viruses to public health.

**Table:** Mutational analysis of HPAI H5N1 viruses from wildlife at Punta Bermeja, Argentina, and other representative HPAI H5N1 strains relative to the goose/Guangdong reference strain

Country, virus strain	Host	Gene segment and mutation position																													
		PB2					PB1			PA				HA			NP					NA				M		NS1			
		T215M	**Q591K#**	**D701N#**	N715T		L378M#	S515A#		R57Q#	T85V	M86I#		H355R	A496S		M222L	Y289F	A428T	R452K		V62I	A81I	S339P#		N87T#		D26K#	E60V	I81T	I129M
Argentina																															
A/South American sea lion/Argentina/RN-PB004/2023*	Mammal	·	**K**	**N**	·		M	A		Q	A	I		·	·		·	·	·	·		·	T	P		T		K	A	·	·
A/South American sea lion/Argentina/RN-PB007/2023*		·	**K**	**N**	·		M	A		Q	A	I		·	·		·	·	·	·		I	T	P		T		K	A	·	·
A/South American sea lion/Argentina/RN-PB011/2023*		·	**K**	**N**	·		M	A		Q	A	I		·	·		·	·	·	·		·	T	P		T		K	A	·	·
A/South American sea lion/Argentina/RN-PB013/2023*		·	**K**	**N**	·		M	A		Q	A	I		·	·		·	·	·	·		·	T	P		T		K	A	·	·
A/South American fur seal/Argentina/RN-PB019/2023*		·	**K**	**N**	·		M	A		Q	A	I		·	·		·	·	·	·		·	T	P		T		K	A	·	·
A/South American tern/Argentina/RN-PB015/2023*	Bird	·	**K**	**N**	·		M	A		Q	A	I		·	·		·	·	·	·		·	T	P		T		K	A	·	·
A/goose/Argentina/140223/ 2023	Bird	?	?	?	?		?	?		?	?	?		.	.		?	?	?	?		.	T	P		?		?	?	?	?
Chile																															
A/Chile/25945/2023	Human	·	**K**	**N**	·		M	·		Q	A	I		·	·		·	·	·	·		·	T	P		T		K	A	·	·
A/sea lion/Arica y Parinacota/240270-1/2023	Mammal	·	·	**N**	·		M	A		Q	A	·		·	·		·	·		·		·	T	P		T		K	A	·	·
A/sanderling/Arica y Parinacota/240265/2023	Bird	·	·	**N**	·		M	A		Q	A	·		·	·		·	·	·	·		·	T	P		T		K	A	·	·
Peru																															
A/sea lion/Peru/TAC-INS-010/2023	Mammal	·	**K**	**N**	·		M	A		Q	A	I		·	·		·	·	·	·		·	T	P		T		K	A	·	·
A/pelican/Peru/PIU-SER019/2022	Bird	·	·	·	·		M	·		·	A	·		·	·		·	·	·	·		·	T	P		T		E	A	·	·
Antartica																															
A/brown skua/Bird Island/128287/2023	Bird	·	·	·	·		M	·		·	A	·		·	·		·	·	·	·		·	T	P		T		E	A	·	·
Brazil																															
A/Thalasseus acuflavidus/EspiritoSanto/1339_N2/2023	Bird	·	·	·	·		M	·		·	A	·		·	·		·	·	·	·		·	T	P		T		E	A	·	·
Uruguay																															
A/black-necked swan/Uruguay/UDELAR-078-M2/2023	Bird	·	·	·	·		M	·		·	A	·		·	·		·	·	·	·		·	T	P		T		E	A	·	·
Ecuador																															
A/Ecuador/6563/2023	Human	?	?	?	?		?	?		?	?	?		.	?		?	?	?	?		?	?	?		?		?	?	?	?
A/wildbird-Fregata-magnificens/Ecuador/IC03-4587/2023	Bird	·	·	·	·		M	·		·	A	·		·	·		·	·	·	·		·	T	P		T		E	A	·	·
Colombia																															
A/duck/Choco/ICA-3501/2022	Bird	·	·	·	·		·	·		·	·	·		·	·		·	·	·	·		·	T	·		·		E	A	·	·
Venezuela																															
A/Pelican/Venezuela/Pel3/2022	Bird	·	·	·	·		·	·		Q	A	·		·	·		·	·	·	·		·	T	·		·		E	A	·	T
United States																															
A/Colorado/18/2022	Human	?	?	?	?		.	.		.	A	.		.	.		?	?	?	?		?	?	?		.		E	A	.	.
A/harbor seal/Washington/23-025991-001/2023	Mammal	·	·	·	·		·	·		·	A	·		·	·		·	·	·	·		·	T	·		·		E	A	·	·
A/chicken/Wyoming/22-009599-002/2022	Bird	·	·	·	·		·	·		·	A	·		·	·		·	·	·	·		·	T	·		·		E	A	·	·
China																															
A/goose/Guangdong/1/96	Bird	T	Q	D	N		L	S		R	T	M		H	S		M	Y	A	R		V	A	S		N		D	E	I	I

*Only 1 strain from the same country and host is used as reference for comparison (whichever had the highest identity to HPAI H5N1 virus from wildlife in Argentina). Asterisks indicate viruses from wildlife at Punta Bermeja, Argentina. Boldface indicates mutations known to be associated with increased virulence, transmission, or mammalian host adaptation. Dots indicate amino acids identical to those in the reference strain. Question marks indicate amino acid positions for which there is no genomic information available. Hash symbols indicate mutations that were already present in viruses infecting sea lions in Peru and Chile but not in the goose/Guangdong reference strain or in viruses from birds and mammals from North America during 2022–2023. HA, hemagglutinin; M, matrix 1 protein; NA, neuraminidase; HPAI, highly pathogenic influenza virus; NP, nucleocapsid protein, NS, nonstructural protein; PA, polymerase acidic protein; PB, polymerase basic protein.

Appendix Additional information for study of high pathogenicity avian influenza A(H5N1) viruses from multispecies outbreak, Argentina, August 2023.
