# Supplementary material for: Highly Pathogenic Avian Influenza A(H5N1) Viruses from Multispecies Outbreak, Argentina, August 2023
Source: Emerg Infect Dis. 2024 Apr;30(4):812–4. doi: 10.3201/eid3004.231725 (PMC10977829; doi:10.3201/eid3004.231725)
Supplement: Appendix — Additional information for study of high pathogenicity avian influenza A(H5N1) viruses from multispecies outbreak, Argentina, August 2023. [file 23-1725-Techapp-s1.pdf]

Article DOI: https://doi.org/10.3201/eid3004.231725

EID cannot ensure accessibility for supplementary materials supplied by authors. Readers who have difficulty accessing supplementary content should contact the authors for assistance.

High-Pathogenicity Avian Influenza A(H5N1) Viruses in Multispecies Outbreak, Argentina, August 2023

Appendix

Details on the laboratory methods employed for the detection and full-genome characterization of HPAI H5N1 viruses in samples from marine mammals and seabirds at Punta Bermeja, Argentina.

Biosecurity

Beach surveys, documentation of clinical cases and sample collection from affected animals were performed by trained veterinarians in full personal protective equipment (hooded tyvek suits, double gloves, N95 respirators, goggles and rubber boots), and under government permits to perform these activities. All swabs collected were placed in cryotubes containing 1 mL of DNA/RNA Shield (Zymo Research, Irvine, CA, USA) for inactivation, then placed in individual ziploc bags (all samples per individual animal), sprayed with 10% bleach, then placed in a second ziploc bag that was stored in a cooler with icepacks. Personal protective equipment and disposable items were discarded as pathogenic waste, and all reusable items (boots, bucket, cooler, knives) were thoroughly washed and scrubbed with water, detergent and a handbrush, and then disinfected with Lysol and 10% bleach before leaving the site. Inactivated samples were transferred to an ultralow freezer (−80°C) within 24 hours after collection, with all samples from a single collection event in double ziploc bags sprayed with 10% bleach. At the laboratory, initial RNA extraction was performed under laminar flow and samples were added to lysis buffer from the QIAamp Viral RNA Mini Kit (Qiagen Inc., Valencia, CA, USA). After waiting the appropriate time for sample inactivation, further steps were performed without laminar flow.

Virus detection

Viral RNA was extracted from 140 µl of suspension from pooled swabs (Appendix Table 1) using a QIAamp Viral RNA Mini Kit Qiagen Inc., Valencia, CA, USA). RNA was eluted in a final volume of 60 µl and stored at –80°C until further use. Viral cDNA was prepared using 15 µl of viral RNA and random hexamers in a final volume of 30 µl using a High Capacity cDNA Archive kit (Applied Biosystems, Foster City, CA, USA). The cDNA was tested for IAV by real-time reverse transcription PCR (RRT-PCR) on an ABI Prism 7500 SDS (Applied Biosystems) using TaqMan Universal PCR Master Mix (Applied Biosystems) directed to the matrix (M) gene (*1*). Quantification cycles (Cq) values were used as a proxy to compare viral RNA load in different samples (Appendix Table 1). Based on average viral RNA loads (summarized in Appendix Table 2), brain samples were selected for further testing. Viral RNA was then extracted from individual brain swabs of four South American sea lions (RN-PB004, RN-PB007, RN-PB011, RN-PB013), one South American fur seal (RN-PB019) and one South American tern (RN-PB015) for further full genome sequencing. The four South American sea lions were randomly selected within age-sex cohorts (i.e., a sub-adult male, a sub-adult female, an adult male, and an adult female). No further analyses were performed on the samples from the great grebe due to low viral RNA yields.

Full genome sequencing

The viral genome was amplified from RNA using a multi-segment one-step RT-PCR with Superscript III high-fidelity RT-PCR kit (Invitrogen, Carlsbad CA) according to manufacturer's instructions using the Opti1 primer set (Opti1-F1, Opti1-F2 and Opti1-R1) as previously described by Zhou and colleagues (*2*). The RT-PCR amplification parameters were: 2 min at 55°C, 60 min at 42°C, and 2 min at 94°C, followed by 5 cycles of (94°C/30 s; 44°C/30 s; 68°C/3.5 min), 26 cycles of (94°C/30 s; 57°C/30 s; 68°C/3.5 min), and a final extension for 10 min at 68°C. Amplicons were visualized on a 1% agarose gel and purified with Agencourt AMPure XP beads (Beckman Coulter, Brea, CA). The concentration of purified amplicons was quantified using the Qubit High Sensitivity dsDNA kit and a Qubit Fluorometer (Invitrogen). The sequencing library preparation was done with the Oxford Nanopore Rapid Barcode library kit SQH-RBK110.96 and loaded on the Mk1c sequencer according to ONT instructions for the R.9 flow cells. Then, real time basecalling was performed with the MinIT to produce fast5 and fastQ files. The automatic real time division into passed and failed reads by the MinIT works as a

quality check, removing reads with quality scores lower than 7. The quality checked reads were demultiplexed and trimmed for adapters and primers, followed by mappings and a final consensus production using Qiagen CLC Genomics Workbench v23.0.2 (Qiagen, Hilden, Germany).

Phylogenetic analysis

Publicly available sequences (as of 9 December 2023) were obtained from GenBank (https://www.ncbi.nlm.nih.gov/genbank/) and the Global Initiative on Sharing All Influenza Data (https://gisaid.org) (*3*). All available sequences from influenza A viruses obtained from samples collected in South America since 1 January 2022 were included, as well as representative samples from other continents (accession codes provided in Appendix Table 3). Sequences were aligned with MAFFT v7.520 (https://mafft.cbrc.jp/alignment/software/) using the FFT-NS-2 algorithm (*4*). Maximum Likelihood trees were built using IQ-TREE v2.2.2.6 (http://www.iqtree.org/), using the integrated ModelFinder for model selection (*5*) and performing 5000 ultra-fast bootstrap repetitions.

References

<eref>1. World Health Organization (WHO). Protocolo del CDC para el RT-PCR en tiempo real para el nuevo subtipo del virus de influenza A(H1N1): Revisión 1 (30 de abril de 2009) [cited 2023 Dec 12]. https://www.paho.org/es/node/36933</eref>

<jrn>2. Zhou B, Donnelly ME, Scholes DT, St George K, Hatta M, Kawaoka Y, et al. Single-reaction genomic amplification accelerates sequencing and vaccine production for classical and Swine origin human influenza a viruses. J Virol. 2009;83:10309–13. 10.1128/JVI.01109-09 PubMed https://doi.org/10.1128/JVI.01109-09</jrn>

<jrn>3. Shu Y, McCauley J. GISAID: Global initiative on sharing all influenza data - from vision to reality. Euro Surveill. 2017;22:30494. 10.2807/1560-7917.ES.2017.22.13.30494 PubMed https://doi.org/10.2807/1560-7917.ES.2017.22.13.30494</jrn>

<jrn>4. Katoh K, Misawa K, Kuma K, Miyata T. MAFFT: a novel method for rapid multiple sequence alignment based on fast Fourier transform. Nucleic Acids Res. 2002;30:3059–66. 10.1093/nar/gkf436 PubMed https://doi.org/10.1093/nar/gkf436</jrn>

<jrn>5. Kalyaanamoorthy S, Minh BQ, Wong TKF, von Haeseler A, Jermiin LS. ModelFinder: fast model selection for accurate phylogenetic estimates. Nat Methods. 2017;14:587–9. 10.1038/nmeth.4285 PubMed https://doi.org/10.1038/nmeth.4285</jrn>

Appendix Table 1. Details of Cq values obtained by RRT-PCR from pooled samples of wildlife collected at Punta Bermeja, Rio Negro, Argentina, in August 2023

Pool	ID	Host species	Age	Sex	Swab	Cq
A	RN-PB002	South American sea lion	Adult	Female	Oronasal	27.24
	RN-PB006	South American sea lion	Sub-adult	Female	Oronasal	
	RN-PB007	South American sea lion	Sub-adult	Female	Oronasal	
	RN-PB013	South American sea lion	Adult	Female	Oronasal	
	RN-PB016	South American sea lion	Adult	Female	Oronasal	
B	RN-PB005	South American sea lion	Adult	Male	Oronasal	26.14
	RN-PB008	South American sea lion	Sub-adult	Male	Oronasal	
	RN-PB011	South American sea lion	Adult	Male	Oronasal	
	RN-PB012	South American sea lion	Adult	Male	Oronasal	
	RN-PB017	South American sea lion	Adult	Male	Oronasal	
	RN-PB018	South American sea lion	Adult	Male	Oronasal	
C	RN-PB003	South American sea lion	Juvenile	Female	Oronasal	25.72
	RN-PB004	South American sea lion	Sub-adult	Male	Oronasal	
	RN-PB009	South American sea lion	Adult	Male	Oronasal	
	RN-PB010	South American sea lion	Juvenile	Female	Oronasal	
	RN-PB019	South American fur seal	Juvenile	Male	Oronasal	
D	RN-PB002	South American sea lion	Adult	Female	Rectal	24.66
	RN-PB006	South American sea lion	Sub-adult	Female	Rectal	
	RN-PB007	South American sea lion	Sub-adult	Female	Rectal	
	RN-PB013	South American sea lion	Adult	Female	Rectal	
	RN-PB016	South American sea lion	Adult	Female	Rectal	
E	RN-PB005	South American sea lion	Adult	Male	Rectal	30.71
	RN-PB008	South American sea lion	Sub-adult	Male	Rectal	
	RN-PB011	South American sea lion	Adult	Male	Rectal	
	RN-PB012	South American sea lion	Adult	Male	Rectal	
	RN-PB017	South American sea lion	Adult	Male	Rectal	
	RN-PB018	South American sea lion	Adult	Male	Rectal	
F	RN-PB003	South American sea lion	Juvenile	Female	Rectal	30.92
	RN-PB004	South American sea lion	Sub-adult	Male	Rectal	
	RN-PB009	South American sea lion	Adult	Male	Rectal	
	RN-PB010	South American sea lion	Juvenile	Female	Rectal	
	RN-PB019	South American fur seal	Juvenile	Male	Rectal	
G	RN-PB002	South American sea lion	Adult	Female	Tracheal	22.97
	RN-PB006	South American sea lion	Sub-adult	Female	Tracheal	
	RN-PB007	South American sea lion	Sub-adult	Female	Tracheal	
	RN-PB013	South American sea lion	Adult	Female	Tracheal	
	RN-PB016	South American sea lion	Adult	Female	Tracheal	
H	RN-PB005	South American sea lion	Adult	Male	Tracheal	19.13
	RN-PB008	South American sea lion	Sub-adult	Male	Tracheal	
	RN-PB011	South American sea lion	Adult	Male	Tracheal	
	RN-PB012	South American sea lion	Adult	Male	Tracheal	
	RN-PB017	South American sea lion	Adult	Male	Tracheal	
	RN-PB018	South American sea lion	Adult	Male	Tracheal	
I	RN-PB003	South American sea lion	Juvenile	Female	Tracheal	23.42
	RN-PB004	South American sea lion	Sub-adult	Male	Tracheal	
	RN-PB009	South American sea lion	Adult	Male	Tracheal	
	RN-PB010	South American sea lion	Juvenile	Female	Tracheal	
	RN-PB019	South American fur seal	Juvenile	Male	Tracheal	
J	RN-PB002	South American sea lion	Adult	Female	Lung	21.58
	RN-PB006	South American sea lion	Sub-adult	Female	Lung	
	RN-PB007	South American sea lion	Sub-adult	Female	Lung	
	RN-PB013	South American sea lion	Adult	Female	Lung	
	RN-PB016	South American sea lion	Adult	Female	Lung	
K	RN-PB005	South American sea lion	Adult	Male	Lung	21.87
	RN-PB008	South American sea lion	Sub-adult	Male	Lung	
	RN-PB011	South American sea lion	Adult	Male	Lung	
	RN-PB012	South American sea lion	Adult	Male	Lung	
	RN-PB017	South American sea lion	Adult	Male	Lung	

Pool	ID	Host species	Age	Sex	Swab	Cq
	RN-PB018	South American sea lion	Adult	Male	Lung	
L	RN-PB003	South American sea lion	Juvenile	Female	Lung	24.31
	RN-PB004	South American sea lion	Sub-adult	Male	Lung	
	RN-PB009	South American sea lion	Adult	Male	Lung	
	RN-PB010	South American sea lion	Juvenile	Female	Lung	
	RN-PB019	South American fur seal	Juvenile	Male	Lung	
M	RN-PB002	South American sea lion	Adult	Female	Brain	19.30
	RN-PB006	South American sea lion	Sub-adult	Female	Brain	
	RN-PB007	South American sea lion	Sub-adult	Female	Brain	
	RN-PB013	South American sea lion	Adult	Female	Brain	
	RN-PB016	South American sea lion	Adult	Female	Brain	
N	RN-PB005	South American sea lion	Adult	Male	Brain	21.84
	RN-PB008	South American sea lion	Sub-adult	Male	Brain	
	RN-PB011	South American sea lion	Adult	Male	Brain	
	RN-PB012	South American sea lion	Adult	Male	Brain	
	RN-PB017	South American sea lion	Adult	Male	Brain	
	RN-PB018	South American sea lion	Adult	Male	Brain	
O	RN-PB003	South American sea lion	Juvenile	Female	Brain	18.87
	RN-PB004	South American sea lion	Sub-adult	Male	Brain	
	RN-PB009	South American sea lion	Adult	Male	Brain	
	RN-PB010	South American sea lion	Juvenile	Female	Brain	
	RN-PB019	South American fur seal	Juvenile	Male	Brain	
P	RN-PB014	South American sea lion	Fetus	Unknown	All	17.56
Q	RN-PB015	South American tern	Juvenile	Male	All	27.74
R	RN-PB020	Great Grebe	Adult	Male	All	35.88

Appendix Table 2. Average Cq values obtained by RRT-PCR from pooled samples collected from different tissues of sea lions at Punta Bermeja, Rio Negro, Argentina, in August 2023

Statistic	Rectal	Oronasal	Lung	Tracheal	Brain
Mean	28.8	26.4	22.6	21.8	20.0
Standard deviation	1.6	1.5	0.8	3.6	2.4

Appendix Table 3. Accession codes for publicly-available gene sequences from GenBank and the Global Initiative on Sharing All Influenza Data used in the phylogenetic and mutation analyses.

Isolate	Accession code
A/Thalasseu acuflavidus/EspiritoSanto/1339 N2/2023 H5N1 Brazil 2023–05–15	OR269884-OR269891
A/Goose/Guangdong/1/96 H5N1 China 1996–01–01	EPI_ISL_1254
A/goose/Argentina/140223/2023 H5N1 Argentina 2023–02–11	EPI_ISL_17527083
A/Chile/25945/2023 H5N1 Chile 2023–03–24	EPI_ISL_17468386
A/sea lion/Tarapaca/240524–2/2023 H5N1 Chile 2023–03–07	EPI_ISL_17885975
A/sea lion/Arica y Parinacota/240270–1/2023 H5N1 Chile 2023–03–01	EPI_ISL_17885976
A/sanderling/Arica y Parinacota/240265/2023 H5N1 Chile 2023–03–03	EPI_ISL_17885978
A/sea lion/Peru/AQP-SER00R/2023 H5N1 Peru 2023–03–06	EPI_ISL_18054510
A/sea lion/Peru/TAC-INS-011/2023 H5N1 Peru 2023–03–07	EPI_ISL_17777532
A/sea lion/Peru/TAC-INS-010/2023 H5N1 Peru 2023–03–07	EPI_ISL_17777531
A/sea lion/Peru/AQP-SER00K/2023 H5N1 Peru 2023–02–07	EPI_ISL_18054509
A/Sea Lion/Peru/LIM-SER036/2023 H5N1 Peru 2023–01–23	EPI_ISL_18054502
A/dolphin/Peru/PIU-SER002/2022 H5N1 Peru 2022–11–22	EPI_ISL_18265431
A/lion/Peru/AIS0554/2023 H5N1 Peru 2023–02–08	EPI_ISL_17805999
A/pelican/Peru/PIU-SER019/2022 H5N1 Peru 2022–11–24	EPI_ISL_18265435
A/Brown skua/Bird Island/128287/2023 H5N1 Antarctica 2023–10–08	EPI_ISL_18439562
A/black-necked swan/Uruguay/UDELAR-078-M2/2023 H5N1 Uruguay 2023–03–15	EPI_ISL_18310957
A/Ecuador/6563/2023 H5N1 Ecuador 2023–01–05	EPI_ISL_17021605
A/wildbird-Fregata-magnificens/Ecuador/IC03–4587/2023 H5N1 Ecuador 2023–01–11	EPI_ISL_17973443
A/duck/Choco/ICA-3501/2022 H5N1 Colombia 2022–10–09	EPI_ISL_17353839
A/Pelican/Venezuela/Pel3/2022 H5N1 Venezuela 2022–11–25	EPI_ISL_16013752
A/Colorado/18/2022 H5N1 USA 2022–04–20	EPI_ISL_12799972
A/skunk/Washington/22–019274–001-original/2022 H5N1 USA 2022–06–07	EPI_ISL_15078254
A/harbor seal/Washington/23–025991–001-original/2023 H5N1 USA 2023–08–25	EPI_ISL_18311025
A/dolphin/Florida/22–025319–002-original/2022 H5N1 USA 2022–03–30	EPI_ISL_15078255
A/chicken/Wyoming/22–009599–002-original/2022 H5N1 USA 2022–03–26	EPI_ISL_13009694
A/Mink/Spain/3691–2 22VIR10586–8/2022 H5N1 Spain 2022–10–18	EPI_ISL_15878541
A/gray seal/Netherlands/30448/2023 H5N1 Netherlands 2023–01–01	EPI_ISL_17672783
A/common dolphin/Wales/040498/2023 H5N1 UK 2023–02–20	EPI_ISL_17465833

Isolate	Accession code
A/Brown skua/Bird Island/128288/2023 H5N1 Antarctica 2023–10–08	EPI_ISL_18439563
A/Brown skua/Bird Island/128289/2023 H5N1 Antarctica 2023–10–08	EPI_ISL_18439564
A/pelican/Peru/PIU-SER028/2022 H5N1 Peru 2022–11–24	EPI_ISL_18265436
A/pelican/Peru/PIU-SER016/2022 H5N1 Peru 2022–11–24	EPI_ISL_18265437
A/Guanay cormorant/Peru/PIU-SER024/2022 H5N1 Peru 2022–11–24	EPI_ISL_18265434
A/Sanderling/Peru/PIU-SER005/2022 H5N1 Peru 2022–11–22	EPI_ISL_18265432
A/pelican/Peru/PIU-SER013/2022 H5N1 Peru 2022–11–23	EPI_ISL_18265433
A/falcon/Peru/A273/2022 H5N1 Peru 2022–12–01	EPI_ISL_17526123
A/owl/Peru/A293/2022 H5N1 Peru 2022–12–01	EPI_ISL_17526122
A/sanderling/Peru/PIU-005/2022 H5N1 Peru 2022–11–22	EPI_ISL_18054501
A/guanay cormorant/Peru/PIU-024/2022 H5N1 Peru 2022–11–24	EPI_ISL_18054500
A/pelican/Peru/PIU-028/2022 H5N1 Peru 2022–11–24	EPI_ISL_18054507
A/pelican/Peru/PIU-019/2022 H5N1 Peru 2022–11–24	EPI_ISL_18054506
A/Pelecanus occidentalis/Venezuela/3S2/2022 H5N1 Venezuela 2022–11–25	EPI_ISL_16854395
A/pelican/Peru/PIU-013/2022 H5N1 Peru 2022–11–23	EPI_ISL_18054504
A/Pelecanus occidentalis/Venezuela/3S1/2022 H5N1 Venezuela 2022–11–25	EPI_ISL_16854394
A/Pelecanus occidentalis/Venezuela/3S4/2022 H5N1 Venezuela 2022–11–25	EPI_ISL_16854397
A/Pelecanus occidentalis/Venezuela/3S3/2022 H5N1 Venezuela 2022–11–25	EPI_ISL_16854396
A/Pelecanus occidentalis/Venezuela/3S6/2022 H5N1 Venezuela 2022–11–25	EPI_ISL_16854399
A/Pelecanus occidentalis/Venezuela/3S5/2022 H5N1 Venezuela 2022–11–25	EPI_ISL_16854398
A/duck/Peru/LAM-INS-014/2023 H5N1 Peru 2023–09–26	EPI_ISL_18497946
A/Pelecanus occidentalis/Venezuela/3S8/2022 H5N1 Venezuela 2022–11–25	EPI_ISL_16854401
A/Pelecanus occidentalis/Venezuela/3S7/2022 H5N1 Venezuela 2022–11–25	EPI_ISL_16854400
A/Pelecanus occidentalis/Venezuela/4S2/2022 H5N1 Venezuela 2022–11–25	EPI_ISL_16854403
A/Pelecanus occidentalis/Venezuela/4S1/2022 H5N1 Venezuela 2022–11–25	EPI_ISL_16854402
A/duck/Peru/LAM-INS-015/2023 H5N1 Peru 2023–09–20	EPI_ISL_18497950
A/Pelecanus occidentalis/Venezuela/4S4/2022 H5N1 Venezuela 2022–11–25	EPI_ISL_16854405
A/Pelecanus occidentalis/Venezuela/4S3/2022 H5N1 Venezuela 2022–11–25	EPI_ISL_16854404
A/Pelecanus occidentalis/Venezuela/4S6/2022 H5N1 Venezuela 2022–11–25	EPI_ISL_16854407
A/Pelecanus occidentalis/Venezuela/4S5/2022 H5N1 Venezuela 2022–11–25	EPI_ISL_16854406
A/Pelecanus occidentalis/Venezuela/4S8/2022 H5N1 Venezuela 2022–11–25	EPI_ISL_16854409
A/Pelecanus occidentalis/Venezuela/4S7/2022 H5N1 Venezuela 2022–11–25	EPI_ISL_16854408
A/american kestrel/Peru/UNMSM-A273/2022 H5N1 Peru 2022–12–01	EPI_ISL_18238548
A/Belcher gull/Peru/UNMSM-A267/2022 H5N1 Peru 2022–12–01	EPI_ISL_18371664
A/Guanay cormorant/Peru/UNMSM-A275/2022 H5N1 Peru 2022–12–01	EPI_ISL_18371665
A/Peruvian booby/Peru/UNMSM-A296/2022 H5N1 Peru 2022–12–01	EPI_ISL_18371666
A/Pelican/Venezuela/Pel4/2022 H5N1 Venezuela 2022–11–25	EPI_ISL_16013753
A/pelican/Peru/PIU-INS-001/2022 H5N1 Peru 2022–11–12	EPI_ISL_16249274
A/chicken/Magdalena/ICA-3503/2022 H5N1 Colombia 2022–11–18	EPI_ISL_17353838
A/belchers gull/Peru/UNMSM-A102/2022 H5N1 Peru 2022–11–01	EPI_ISL_18238610
A/duck/Peru/CAL-INS-013/2023 H5N1 Peru 2023–07–24	EPI_ISL_18217104
A/peruvian pelican/Peru/UNMSM-A074/2022 H5N1 Peru 2022–11–01	EPI_ISL_18238608
A/belchers gull/Peru/UNMSM-A267/2022 H5N1 Peru 2022–12–01	EPI_ISL_18238609
A/western barn owl/Peru/UNMSM-A293/2022 H5N1 Peru 2022–12–01	EPI_ISL_18238622
A/peruvian pelican/Peru/UNMSM-A106/2022 H5N1 Peru 2022–11–01	EPI_ISL_18238607
A/owl/Peru/23–005629–001-original/2022 H5N1 Peru 2022–11–11	EPI_ISL_17660073
A/chicken/Peru/23–005607–003-original/2022 H5N1 Peru 2022–11–11	EPI_ISL_17660072
A/brown booby/Peru/23–005629–003-original/2022 H5N1 Peru 2022–11–11	EPI_ISL_17660074
A/chicken/Peru/23–005607–002-original/2022 H5N1 Peru 2022–11–11	EPI_ISL_17660071
A/chicken/Peru/23–005607–001-original/2022 H5N1 Peru 2022–11–11	EPI_ISL_17660070
A/Pelecanus/Peru/VFAR-140/2022 H5N1 Peru 2022–12–01	EPI_ISL_17099964
A/wildbird-Fregata-magnificens/Ecuador/IC06–4590/2023 H5N1 Ecuador 2023–01–11	EPI_ISL_17973458
A/Gull/Chile/7023–3/2022 H5N1 Chile 2022–12–07	EPI_ISL_17011958
A/Gull/Chile/7023–2/2022 H5N1 Chile 2022–12–07	EPI_ISL_17011964
A/turkey vulture/Valparaiso/230187–1/2022 H5N1 Chile 2022–12–31	EPI_ISL_17885968
A/domestic duck/Maule/240466–1/2023 H5N1 Chile 2023–03–07	EPI_ISL_18005779
A/heron/Antofagasta/228705–3/2022 H5N1 Chile 2022–12–20	EPI_ISL_17885969
A/turkey vulture/Antofagasta/228252–1/2022 H5N1 Chile 2022–12–17	EPI_ISL_17885970
A/heron/Antofagasta/228705–2/2022 H5N1 Chile 2022–12–20	EPI_ISL_17885971
A/kelp gull/Maule/239349/2023 H5N1 Chile 2023–03–01	EPI_ISL_17885972
A/domestic duck/Araucania/239189–3/2023 H5N1 Chile 2023–02–28	EPI_ISL_18005783
A/tern/Maule/238507/2023 H5N1 Chile 2023–02–23	EPI_ISL_17885973
A/domestic duck/Araucania/241914–2/2023 H5N1 Chile 2023–03–14	EPI_ISL_18005780
A/domestic duck/Araucania/240481–2/2023 H5N1 Chile 2023–03–07	EPI_ISL_18005781
A/black crowned night heron/Antofagasta/228705–2/2022 H5N1 Chile 2022–12–20	EPI_ISL_18005786
A/backyard chicken/Uruguay/UDELAR-040-M5/2023 H5N1 Uruguay 2023–03–03	EPI_ISL_18310942
A/black crowned night heron/Antofagasta/228705–3/2022 H5N1 Chile 2022–12–20	EPI_ISL_18005784
A/sanderling/Arica y Parinacota/230758–1/2022 H5N1 Chile 2022–12–30	EPI_ISL_17885980
A/pelican/Valparaiso/234040/2023 H5N1 Chile 2023–01–25	EPI_ISL_17885982

Isolate	Accession code
A/pelican/Valparaiso/233450–1/2023 H5N1 Chile 2023–01–23	EPI_ISL_17885983
A/chicken/Araucania/239569–2/2023 H5N1 Chile 2023–03–02	EPI_ISL_17885952
A/elegant tern/Tarapaca/229133–1/2022 H5N1 Chile 2022–12–23	EPI_ISL_17885953
A/chicken/Araucania/239569–1/2023 H5N1 Chile 2023–03–02	EPI_ISL_17885954
A/elegant tern/Arica y Parinacota/229476–1/2022 H5N1 Chile 2022–12–24	EPI_ISL_17885955
A/pelican/Valparaiso/233091–2/2023 H5N1 Chile 2023–01–20	EPI_ISL_17885956
A/duck/Maule/240466–1/2023 H5N1 Chile 2023–03–07	EPI_ISL_17885957
A/chicken/Araucania/239189–2/2023 H5N1 Chile 2023–02–28	EPI_ISL_17885958
A/duck/Araucania/241914–2/2023 H5N1 Chile 2023–03–14	EPI_ISL_17885959
A/whimbrel/Coquimbo/239964/2023 H5N1 Chile 2023–03–05	EPI_ISL_17885960
A/duck/Araucania/240481–2/2023 H5N1 Chile 2023–03–07	EPI_ISL_17885961
A/turkey/Nuble/241568–1/2023 H5N1 Chile 2023–03–10	EPI_ISL_17885962
A/duck/Araucania/239189–3/2023 H5N1 Chile 2023–02–28	EPI_ISL_17885963
A/turkey/Nuble/240489–1/2023 H5N1 Chile 2023–03–07	EPI_ISL_17885964
A/chiloe wigeon/OHiggins/240893–2/2023 H5N1 Chile 2023–03–09	EPI_ISL_17885965
A/turkey/Araucania/241892–3/2023 H5N1 Chile 2023–03–14	EPI_ISL_17885966
A/blackish oystercatcher/OHiggins/240628/2023 H5N1 Chile 2023–03–06	EPI_ISL_17885967
A/backyard chicken/Uruguay/UDELAR-040-M7/2023 H5N1 Uruguay 2023–03–03	EPI_ISL_18310967
A/backyard chicken/Uruguay/UDELAR-047-M1/2023 H5N1 Uruguay 2023–03–06	EPI_ISL_18310966
A/backyard chicken/Uruguay/UDELAR-047-M3/2023 H5N1 Uruguay 2023–03–06	EPI_ISL_18310965
A/backyard chicken/Uruguay/UDELAR-124-M1/2023 H5N1 Uruguay 2023–04–16	EPI_ISL_18310964
A/backyard chicken/Uruguay/UDELAR-127-M1/2023 H5N1 Uruguay 2023–04–17	EPI_ISL_18310963
A/backyard chicken/Uruguay/UDELAR-127-M4/2023 H5N1 Uruguay 2023–04–17	EPI_ISL_18310962
A/backyard chicken/Uruguay/UDELAR-144-M3/2023 H5N1 Uruguay 2023–05–03	EPI_ISL_18310961
A/backyard duck/Uruguay/UDELAR-124-M3/2023 H5N1 Uruguay 2023–04–16	EPI_ISL_18310960
A/chicken/Bolivar/ICA-3500/2022 H5N1 Colombia 2022–11–08	EPI_ISL_17353507
A/chicken/Choco/ICA-3502/2022 H5N1 Colombia 2022–11–13	EPI_ISL_17353508
A/chicken/Choco/ICA-3504/2022 H5N1 Colombia 2022–11–01	EPI_ISL_17353509
A/chicken/Cordoba/ICA-3499/2022 H5N1 Colombia 2022–11–26	EPI_ISL_17353510
A/pelican/Valparaiso/233447–2/2023 H5N1 Chile 2023–01–21	EPI_ISL_17885985
A/whimbrel/Valparaiso/239946/2023 H5N1 Chile 2023–03–05	EPI_ISL_17885987
A/backyard turkey/Uruguay/UDELAR-124-M6/2023 H5N1 Uruguay 2023–04–16	EPI_ISL_18310959
A/black-necked swan/Uruguay/UDELAR-014-M3/2023 H5N1 Uruguay 2023–02–18	EPI_ISL_18310958
A/peruvian booby/Peru/LIM-INS-012/2023 H5N1 Peru 2023–04–12	EPI_ISL_17777533
A/guanay cormorant/Peru/CAL-INS-009/2023 H5N1 Peru 2023–03–17	EPI_ISL_17777530
A/peruvian booby/Peru/CAL-INS-008/2023 H5N1 Peru 2023–03–17	EPI_ISL_17777529
A/peruvian booby/Peru/CAL-INS-007/2023 H5N1 Peru 2023–03–17	EPI_ISL_17777528
A/gull/Peru/LIM-INS-006/2023 H5N1 Peru 2023–03–16	EPI_ISL_17777527
A/peruvian booby/Peru/LIM-INS-005/2023 H5N1 Peru 2023–03–20	EPI_ISL_17777526
A/peruvian booby/Peru/LIM-INS-004/2023 H5N1 Peru 2023–04–03	EPI_ISL_17777525
A/Peruvian pelican/Chile/C61740/2022 H5N1 Chile 2022–12–02	EPI_ISL_16891401
A/black skimmer/Chile/C61962/2022 H5N1 Chile 2022–12–02	EPI_ISL_16891402
A/wildbird-Sula-nebouxii/Ecuador/7607/2023 H5N1 Ecuador 2023–05–18	EPI_ISL_18137626
A/wildbird-Sula-nebouxii/Ecuador/7611/2023 H5N1 Ecuador 2023–05–18	EPI_ISL_18137671
A/pelican/Peru/A074/2022 H5N1 Peru 2022–11–01	EPI_ISL_17477219
A/gull/Peru/A267/2022 H5N1 Peru 2022–12–01	EPI_ISL_17477222
A/gull/Peru/A102/2022 H5N1 Peru 2022–11–01	EPI_ISL_17477220
A/pelican/Peru/A106/2022 H5N1 Peru 2022–11–01	EPI_ISL_17477221
A/chicken/Peru/LIM-INS-003/2022 H5N1 Peru 2022–11–29	EPI_ISL_16249730
A/chicken/Ecuador/04/2022 H5N1 Ecuador 2022–11–25	EPI_ISL_16161675
A/chicken/Ecuador/03/2022 H5N1 Ecuador 2022–11–25	EPI_ISL_16161673
A/black skimmer/Maule/240379/2023 H5N1 Chile 2023–03–06	EPI_ISL_17885849
A/chicken/Ecuador/02/2022 H5N1 Ecuador 2022–11–25	EPI_ISL_16157545
A/chicken/OHiggins/241252–1/2023 H5N1 Chile 2023–03–12	EPI_ISL_17885872
A/chicken/Nuble/241681–1/2023 H5N1 Chile 2023–03–10	EPI_ISL_17885873
A/avian/Peru/AISA0451/2022 H5N1 Peru 2022–11–22	EPI_ISL_17805998
A/chicken/Nuble/241557–1/2023 H5N1 Chile 2023–03–10	EPI_ISL_17885874
A/avian/Peru/AISA0446/2022 H5N1 Peru 2022–11–22	EPI_ISL_17805997
A/chicken/Peru/AIS0549/2022 H5N1 Peru 2022–12–22	EPI_ISL_17805996
A/chicken/Peru/AIS0550/2022 H5N1 Peru 2022–12–12	EPI_ISL_17805995
A/chicken/Nuble/240684/2023 H5N1 Chile 2023–03–08	EPI_ISL_17885876
A/chicken/Peru/AIS0548/2022 H5N1 Peru 2022–12–22	EPI_ISL_17805994
A/chicken/Nuble/240155/2023 H5N1 Chile 2023–03–06	EPI_ISL_17885877
A/chicken/Nuble/239136/2023 H5N1 Chile 2023–02–27	EPI_ISL_17885878
A/chicken/Peru/AIS0547/2022 H5N1 Peru 2022–12–28	EPI_ISL_17805993
A/chicken/Peru/LAM-INS-002/2022 H5N1 Peru 2022–11–28	EPI_ISL_16249681
A/chicken/Peru/AIS0545/2022 H5N1 Peru 2022–12–03	EPI_ISL_17805992
A/chicken/Peru/AIS0544/2022 H5N1 Peru 2022–12–03	EPI_ISL_17805991
A/chicken/Peru/AIS0543/2022 H5N1 Peru 2022–12–01	EPI_ISL_17805990

Isolate	Accession code
A/chicken/Peru/AIS0542/2022 H5N1 Peru 2022–12–01	EPI_ISL_17805989
A/chicken/Peru/AIS0540/2022 H5N1 Peru 2022–11–28	EPI_ISL_17805988
A/chicken/Peru/AIS0539/2022 H5N1 Peru 2022–11–18	EPI_ISL_17805987
A/chicken/Peru/AIS0546/2022 H5N1 Peru 2022–12–18	EPI_ISL_17805986
A/pelican/Peru/AISA0464/2022 H5N1 Peru 2022–11–22	EPI_ISL_17806003
A/goose/Araucania/239189–1/2023 H5N1 Chile 2023–02–28	EPI_ISL_17885869
A/pelican/Peru/AIS0541/2022 H5N1 Peru 2022–11–16	EPI_ISL_17806002
A/chicken/OHiggins/241252–6/2023 H5N1 Chile 2023–03–12	EPI_ISL_17885870
A/chicken/Peru/AIS0551/2022 H5N1 Peru 2022–12–12	EPI_ISL_17806001
A/pelican/Peru/AIS0538/2022 H5N1 Peru 2022–11–10	EPI_ISL_17806000
A/chicken/OHiggins/241252–3/2023 H5N1 Chile 2023–03–12	EPI_ISL_17885871
A/pelican/OHiggins/233721–1/2023 H5N1 Chile 2023–01–24	EPI_ISL_17885912
A/pelican/OHiggins/233663–1/2023 H5N1 Chile 2023–01–24	EPI_ISL_17885915
A/pelican/Nuble/233947–2/2023 H5N1 Chile 2023–01–26	EPI_ISL_17885918
A/pelican/Tarapaca/227436–2/2022 H5N1 Chile 2022–12–09	EPI_ISL_17885942
A/pelican/Antofagasta/228244–2/2022 H5N1 Chile 2022–12–16	EPI_ISL_17885944
A/chicken/Araucania/241914–1/2023 H5N1 Chile 2023–03–14	EPI_ISL_17885945
A/chicken/Araucania/241892–2/2023 H5N1 Chile 2023–03–14	EPI_ISL_17885946
A/humboldt penguin/Tarapaca/238744–2/2023 H5N1 Chile 2023–02–23	EPI_ISL_17885947
A/humboldt penguin/Coquimbo/239590/2023 H5N1 Chile 2023–02–28	EPI_ISL_17885948
A/chicken/Araucania/240481–1/2023 H5N1 Chile 2023–03–07	EPI_ISL_17885949
A/great egret/Araucania/240518/2023 H5N1 Chile 2023–03–07	EPI_ISL_17885950
A/gray gull/Tarapaca/232825–1/2023 H5N1 Chile 2023–01–18	EPI_ISL_17885951
A/pelican/Maule/231155–2/2023 H5N1 Chile 2023–01–09	EPI_ISL_17885921
A/pelican/Coquimbo/231946–1/2023 H5N1 Chile 2023–01–14	EPI_ISL_17885922
A/pelican/Coquimbo/230310–1/2023 H5N1 Chile 2023–01–02	EPI_ISL_17885923
A/pelican/Atacama/230158–2/2022 H5N1 Chile 2022–12–30	EPI_ISL_17885924
A/pelican/Atacama/230158–1/2022 H5N1 Chile 2022–12–30	EPI_ISL_17885925
A/pelican/Atacama/229450–2/2022 H5N1 Chile 2022–12–26	EPI_ISL_17885926
A/pelican/Atacama/229424–2/2022 H5N1 Chile 2022–12–22	EPI_ISL_17885927
A/pelican/Antofagasta/228318–1/2022 H5N1 Chile 2022–12–16	EPI_ISL_17885928
A/pelican/Antofagasta/228272–1/2022 H5N1 Chile 2022–12–18	EPI_ISL_17885929
A/pelican/Valparaiso/233091–1/2023 H5N1 Chile 2023–01–20	EPI_ISL_17885930
A/pelican/Antofagasta/228246–3/2022 H5N1 Chile 2022–12–15	EPI_ISL_17885931
A/pelican/Valparaiso/233418–1/2023 H5N1 Chile 2023–01–20	EPI_ISL_17885932
A/pelican/Antofagasta/228246–2/2022 H5N1 Chile 2022–12–15	EPI_ISL_17885934
A/south american sea lion/Peru/AQP-SER00R/2023 H5N1 Peru 2023–03–06	EPI_ISL_18265422
A/sea lion/Peru/AQP-SER00B/2023 H5N1 Peru 2023–01–25	EPI_ISL_18054508

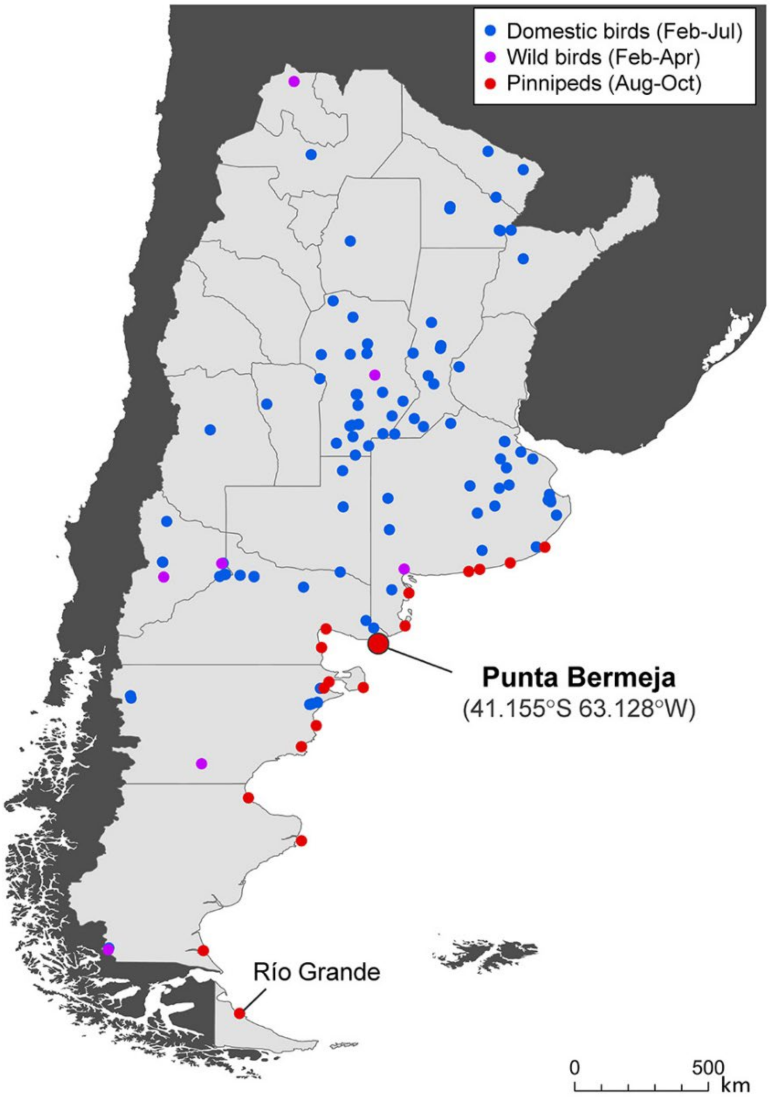

Appendix Figure 1. Location of Punta Bermeja and other HPAI H5N1 detections in pinnipeds since August 2023 (red dots) and HPAI H5N1 detections in poultry (blue dots) and wild birds (purple dots) in Argentina.

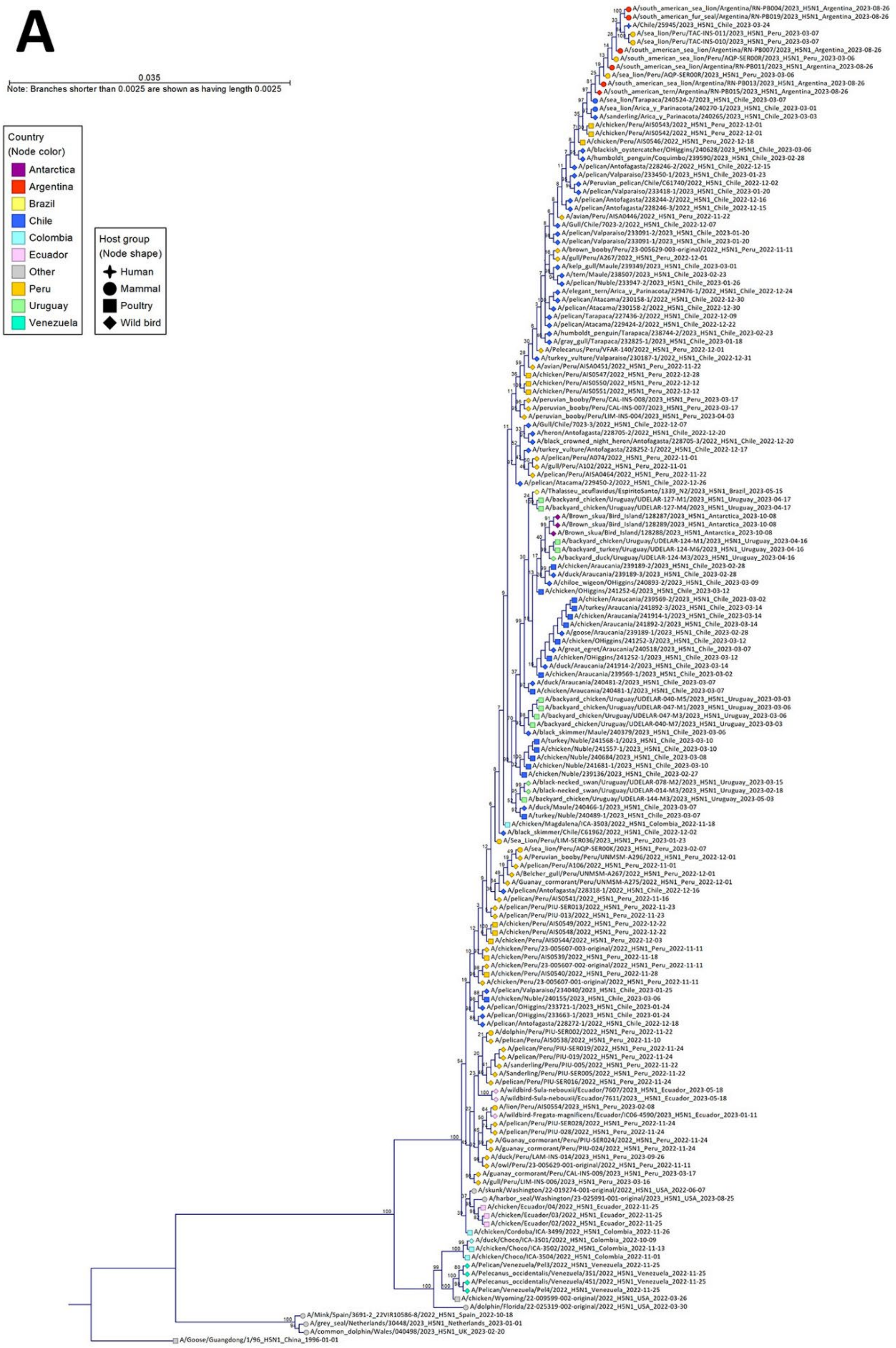
A
0.035
Note: Branches shorter than 0.0025 are shown as having length 0.0025
Country
(Node color)
Antarctica
Argentina
Brazil
Chile
Colombia
Ecuador
Other
Peru
Uruguay
Venezuela
Host group
(Node shape)
Human
Mammal
Poultry
Wild bird

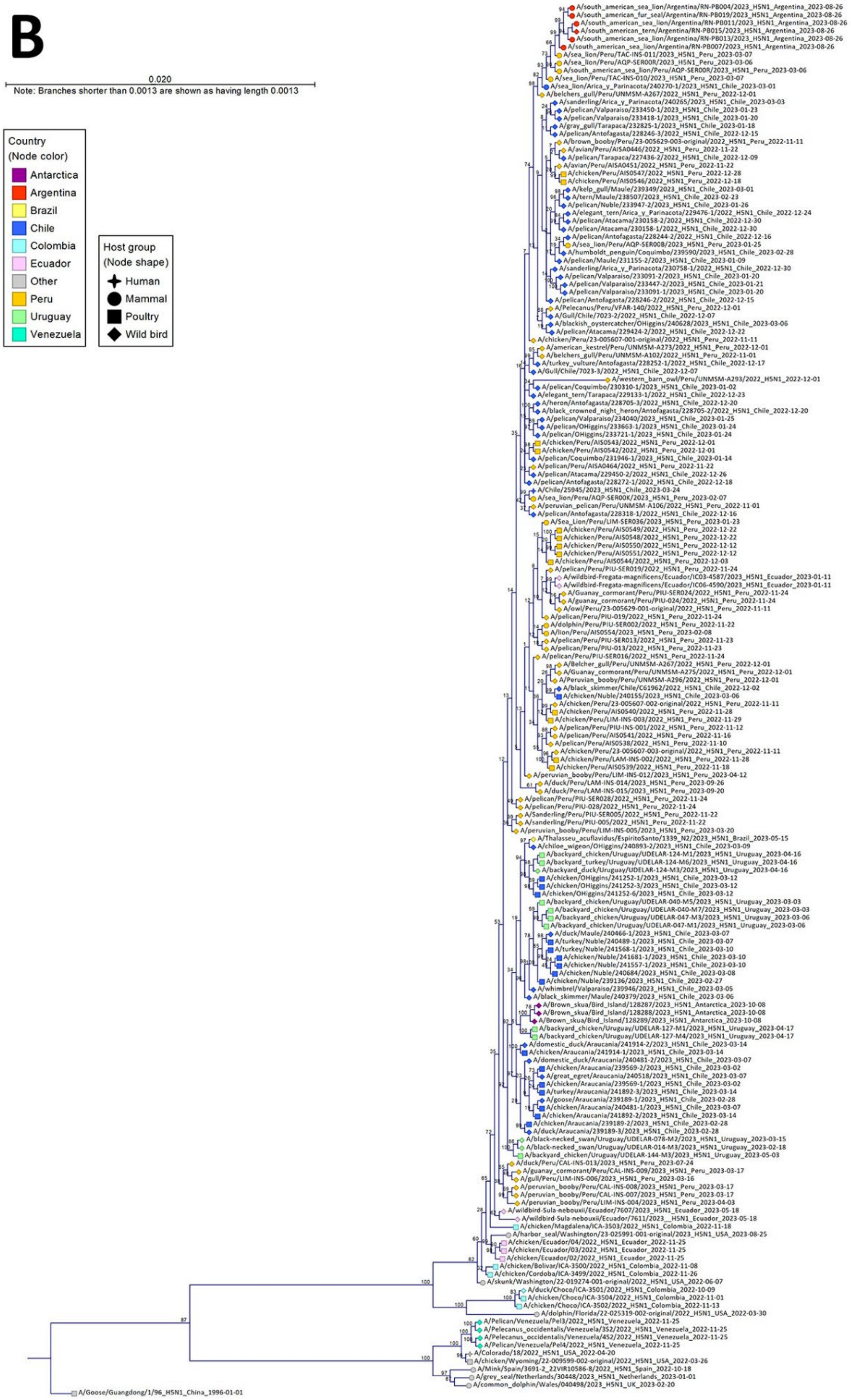
B
0.020
Note: Branches shorter than 0.0013 are shown as having length 0.0013
Country
(Node color)
Antarctica
Argentina
Brazil
Chile
Colombia
Ecuador
Other
Peru
Uruguay
Venezuela
Host group
(Node shape)
Human
Mammal
Poultry
Wild bird

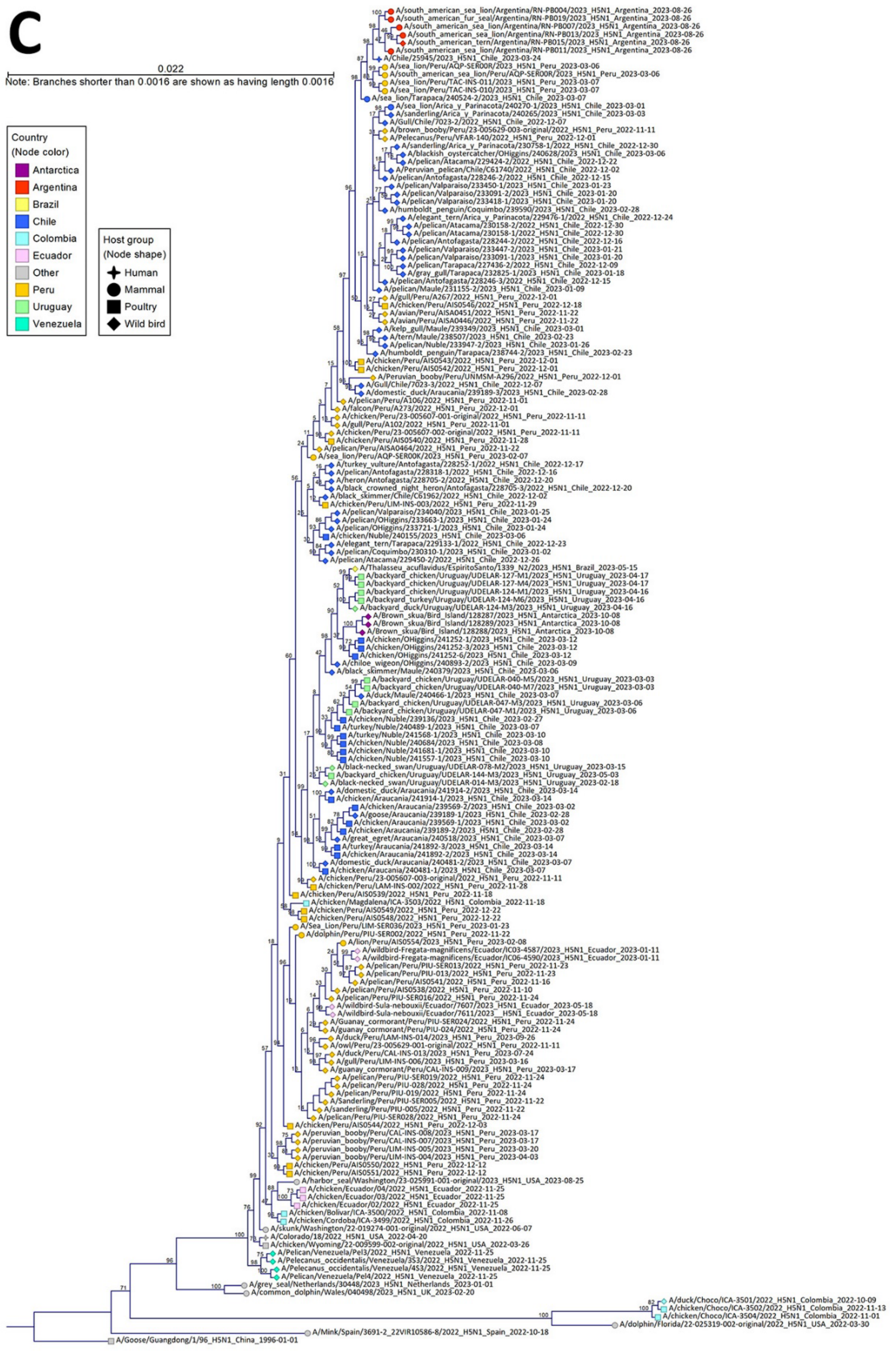
C
0.022
Note: Branches shorter than 0.0016 are shown as having length 0.0016
Country (Node color)
Antarctica
Argentina
Brazil
Chile
Colombia
Ecuador
Other
Peru
Uruguay
Venezuela
Host group (Node shape)
Human
Mammal
Poultry
Wild bird

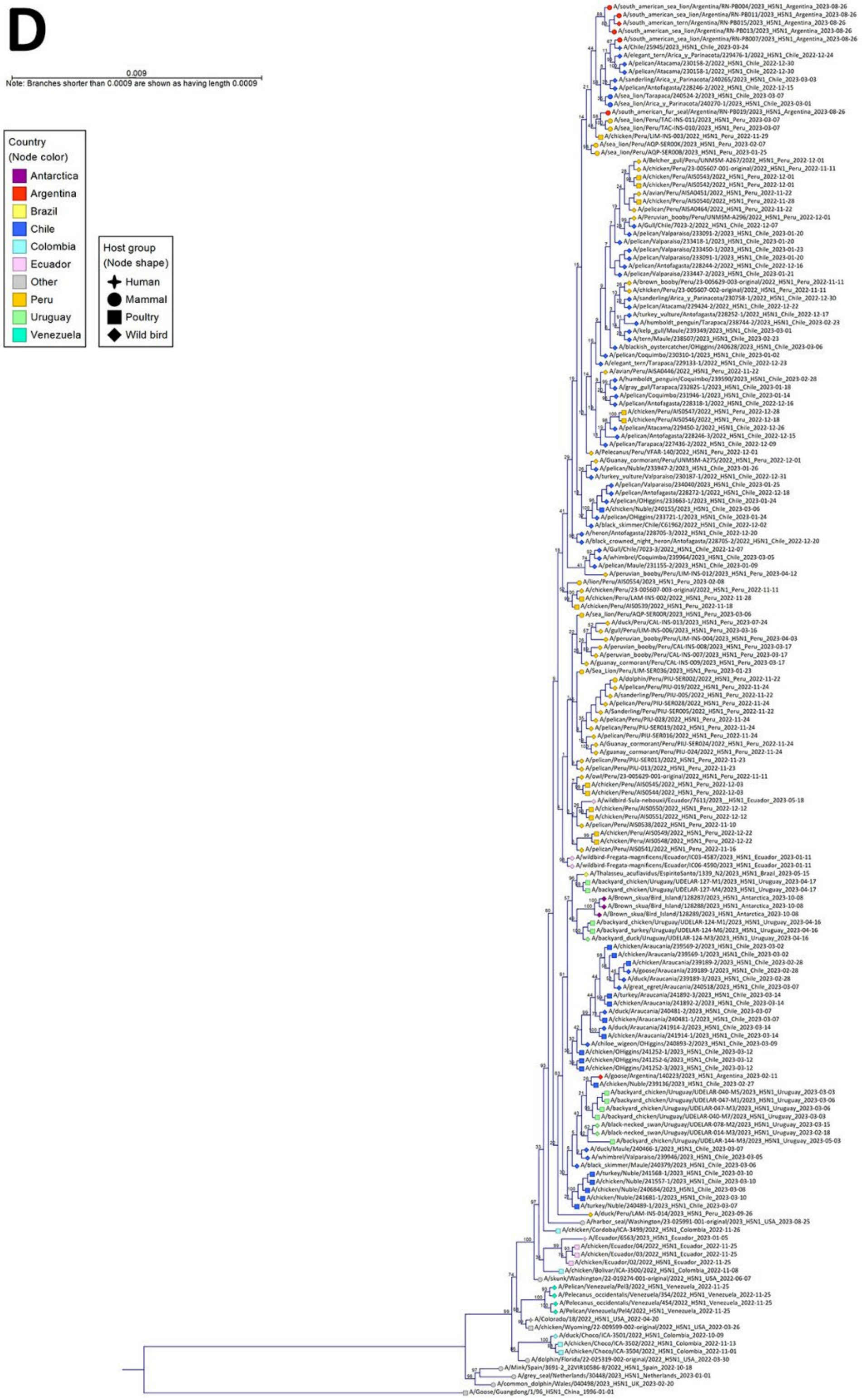
D
0.009
Note: Branches shorter than 0.0009 are shown as having length 0.0009
Country
(Node color)
Antarctica
Argentina
Brazil
Chile
Colombia
Ecuador
Other
Peru
Uruguay
Venezuela
Host group
(Node shape)
Human
Mammal
Poultry
Wild bird

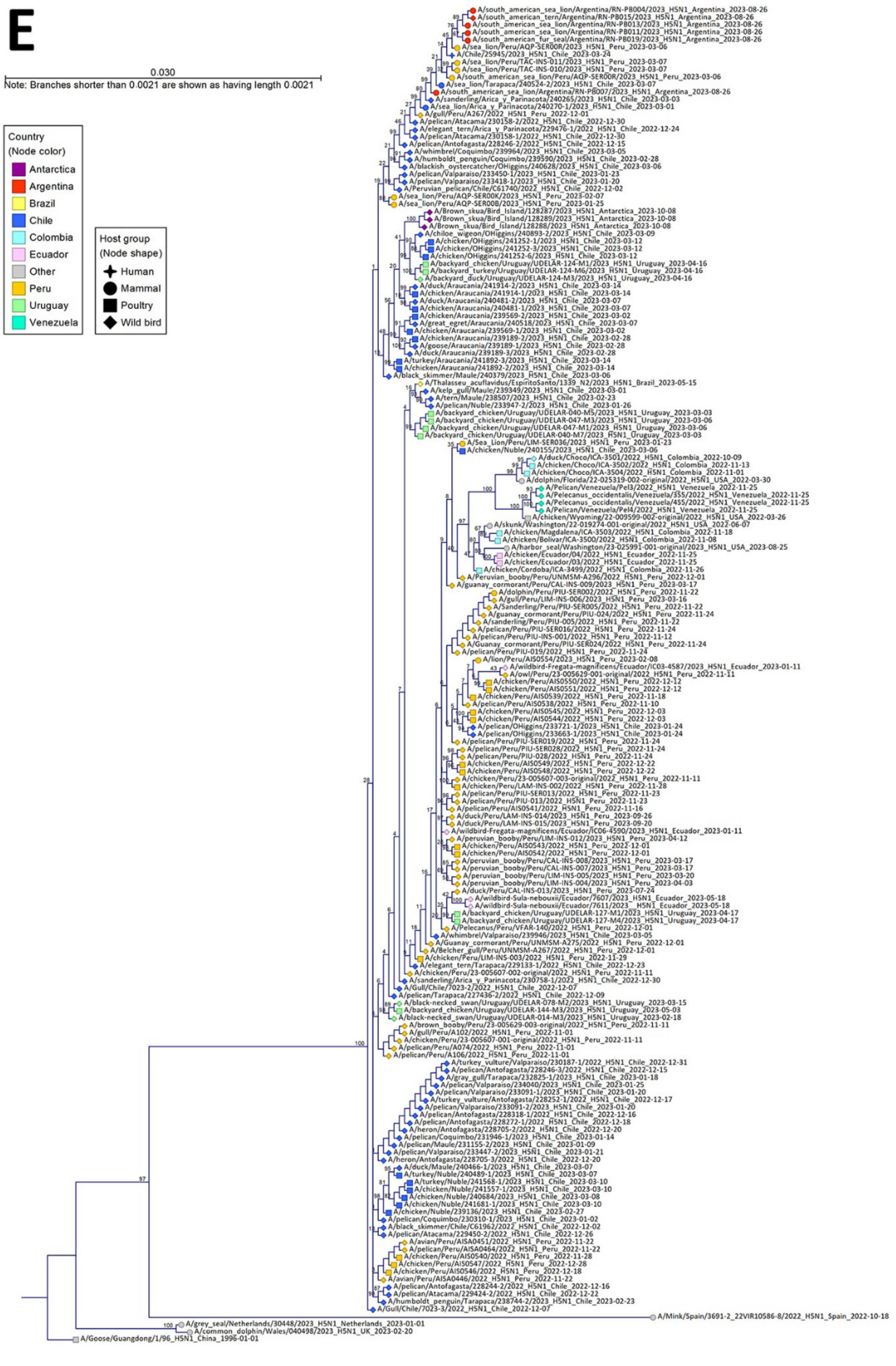
E
0.030
Note: Branches shorter than 0.0021 are shown as having length 0.0021
Country
(Node color)
Antarctica
Argentina
Brazil
Chile
Colombia
Ecuador
Other
Peru
Uruguay
Venezuela
Host group
(Node shape)
Human
Mammal
Poultry
Wild bird

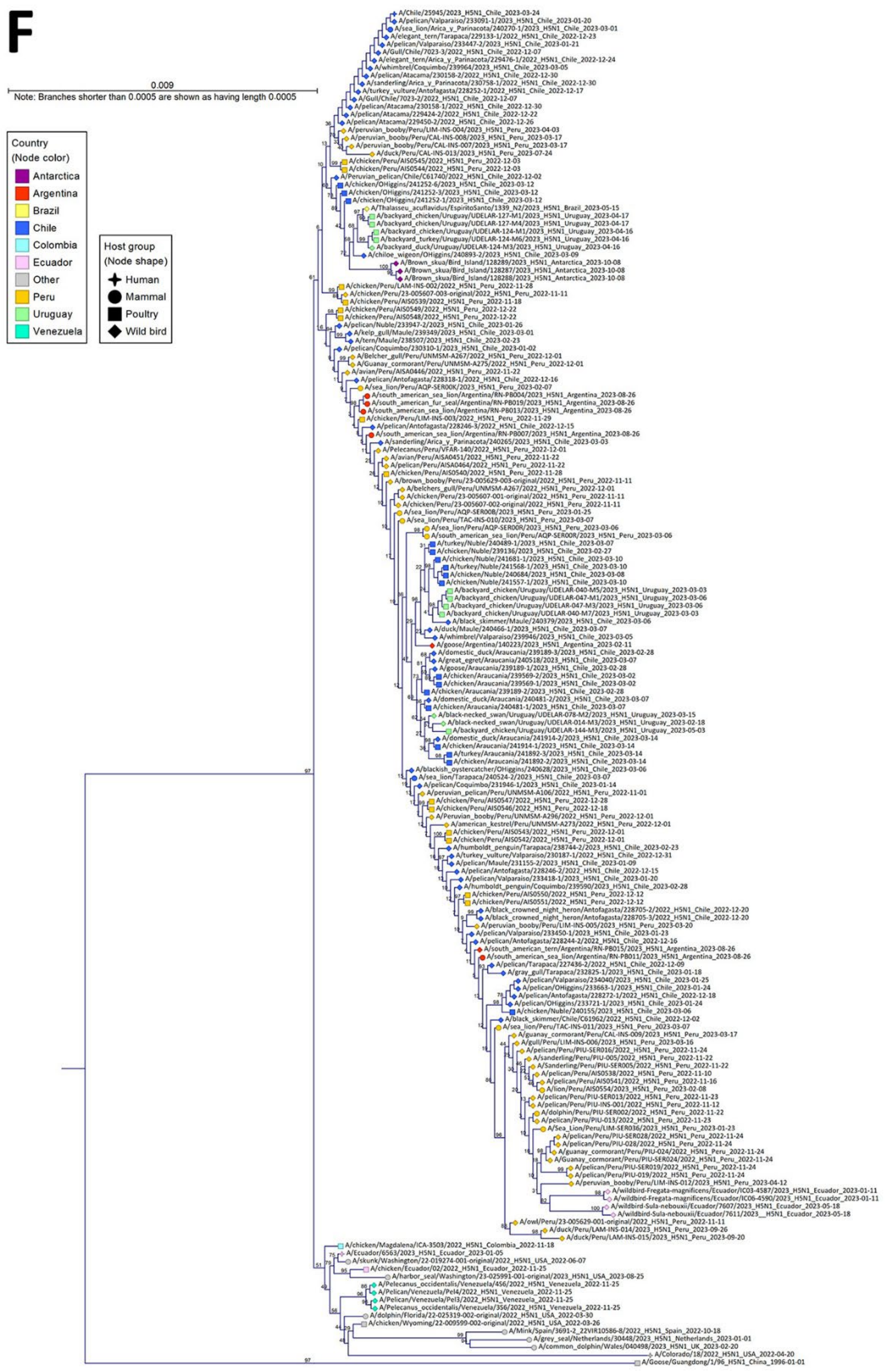
F
0.009
Note: Branches shorter than 0.0005 are shown as having length 0.0005
Country
(Node color)
Antarctica
Argentina
Brazil
Chile
Colombia
Ecuador
Other
Peru
Uruguay
Venezuela
Host group
(Node shape)
Human
Mammal
Poultry
Wild bird

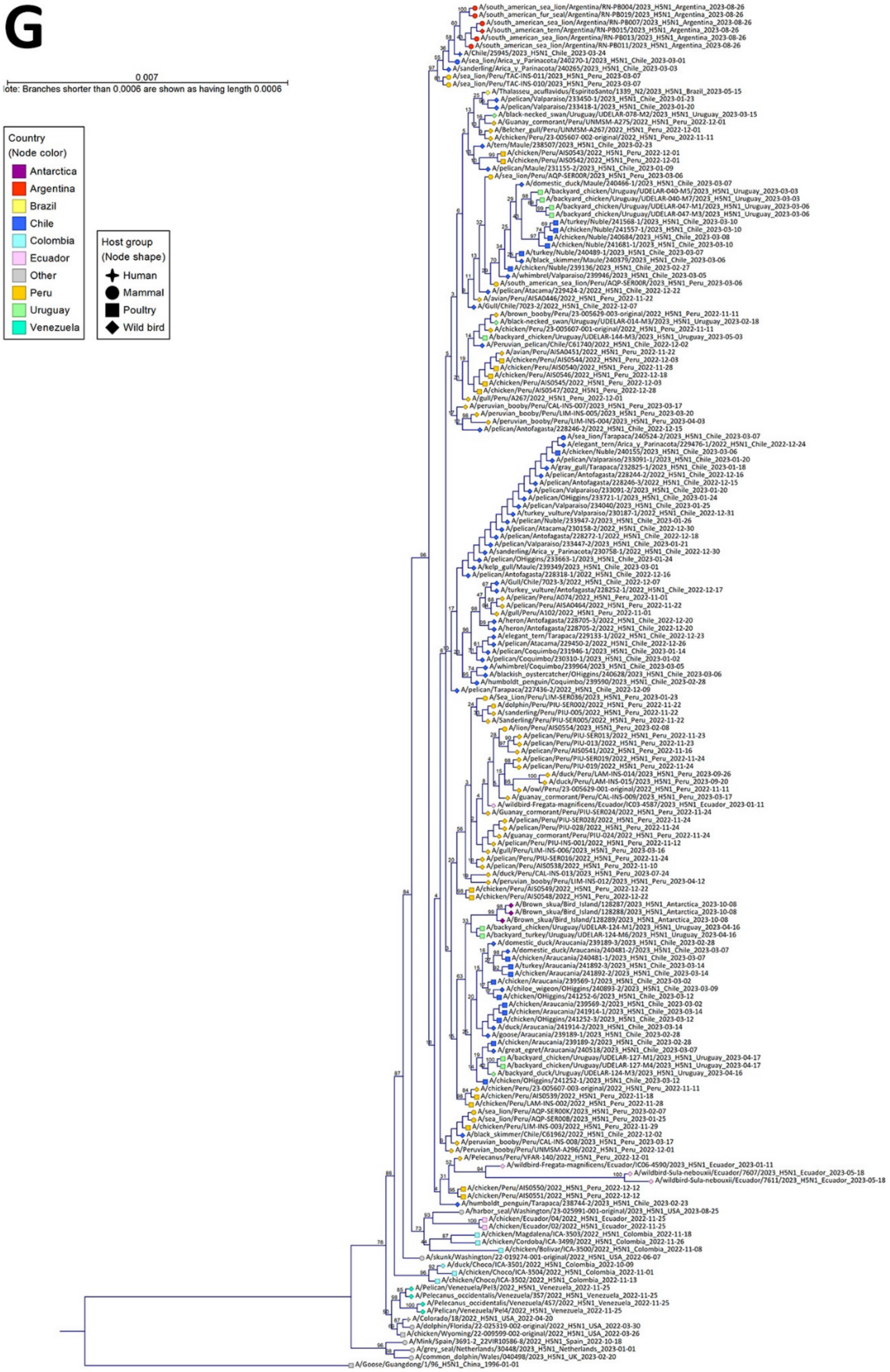
G
0.007
Note: Branches shorter than 0.0006 are shown as having length 0.0006
Country
(Node color)
Antarctica
Argentina
Brazil
Chile
Colombia
Ecuador
Other
Peru
Uruguay
Venezuela
Host group
(Node shape)
Human
Mammal
Poultry
Wild bird

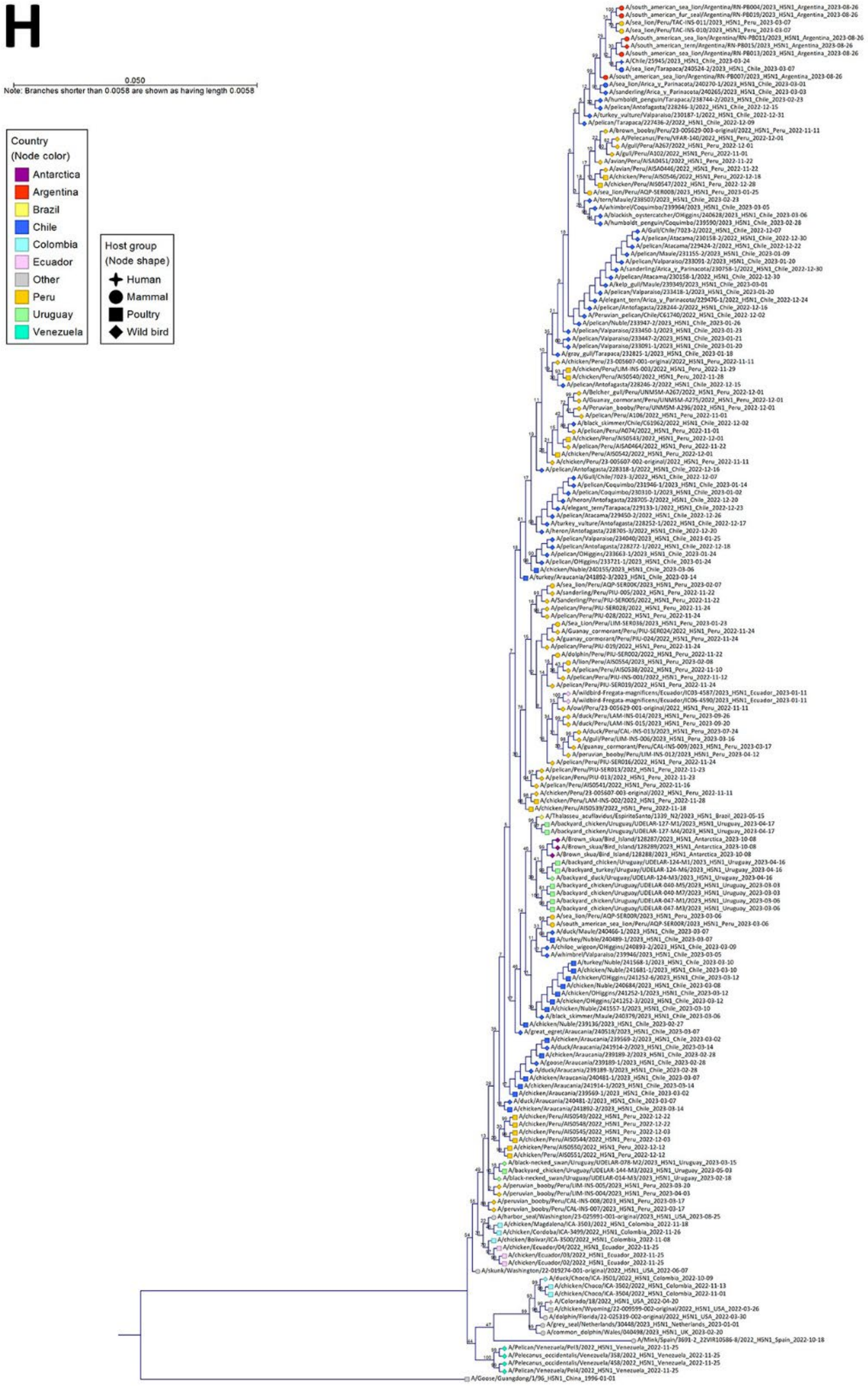

Appendix Figure 2. Maximum likelihood tree for PB2, PB1, PA, HA, NP, NA, MP, and NS gene segments of HPAI H5N1 strains from Punta Bermeja, Argentina, in relation to all HPAI H5N1 strains from South America and a few strains from North America and Europe to show viral evolution in the southern hemisphere. Node shape represents host group, and node color (and bars adjacent to trees) represents the region/country. Branch lengths are drawn proportionally to the extent of changes (scale-bars are shown). Values adjacent to nodes represent bootstrap support.
